# Labeling surface proteins with high specificity: Intrinsic limitations of phosphopantetheinyl transferase systems

**DOI:** 10.1371/journal.pone.0226579

**Published:** 2019-12-19

**Authors:** Jakob C. Stüber, Andreas Plückthun

**Affiliations:** Department of Biochemistry, University of Zurich, Winterthurerstrasse, Zurich, Switzerland; Berlin Institute of Technology, GERMANY

## Abstract

**Objective:**

Fluorescent labeling of specific cell-surface proteins enables a manifold of techniques to study their function in health and disease. A frequently cited family of methods employs phosphopantetheinyl transferases (PPTases) to attach probes, provided as conjugates of Coenzyme A. This method appears attractive, as only short peptide tags genetically fused to the protein of interest are needed as conjugation sites. Here, we describe observations we made when evaluating such protocols for delicate single-molecule applications where we require a particular combination of dyes, low background binding or low labeling of other proteins, and a high degree of labeling.

**Results:**

When we tested a PPTase-acceptor peptide couple with several experimental protocols and various CoA conjugates for labeling of a protein on the cell surface, we noticed substantial non-specific labeling. For the first time, we provide here a quantification of the non-specific fraction of the signals obtained using appropriate controls. We further present evidence that this background is due to CoA-dye conjugates entering the cell, where they may be covalently attached to endogenous proteins. However, when studying cell-surface proteins, most fluorescent readouts require that labeling is strictly limited to the protein of interest located at the cell surface. While such data have so far been missing in the literature, they suggest that for applications where labeling of unwanted molecules would affect the conclusions, researchers need to be aware of this potential non-specificity of PPTase methods when selecting a labeling strategy. We show, again by quantitative comparison, that the HaloTag is a viable alternative.

## Introduction

Techniques based on fluorescence are pivotal for advancing our understanding of cell surface protein dynamics, interactions, and regulation. While fluorescent proteins, genetically fused to the protein of interest, have had an enormous impact in biomedical research, they are not suited for all applications because of their photophysical properties, which are often inferior to those of organic dyes [1]. This is particularly important in single-molecule experiments. Furthermore, in studies examining the biosynthesis and/or internalization and degradation of cell surface proteins, it is frequently necessary to selectively label the surface fraction, either at equilibrium or in a time-dependent manner.

Intracellular fluorescence must usually be avoided in such studies, because most optical techniques will also record these signals, leading to a convolution with the signals actually originating from the membrane-associated proteins of interest. Also, biosynthetic intermediates or degradation products of fusion proteins, which are fluorescent but otherwise non-functional, could interfere with the analysis. Furthermore, the dimerization-propensity of many fluorescent proteins may perturb the experimental system by artifactually introducing di- or oligomerization of the membrane protein under study [2]. Thus, methods to attach fluorophores (or, more generally speaking, chemical entities) to proteins in their native environment—on live cells—have gained importance in life science research [3].

**Fig 1:**
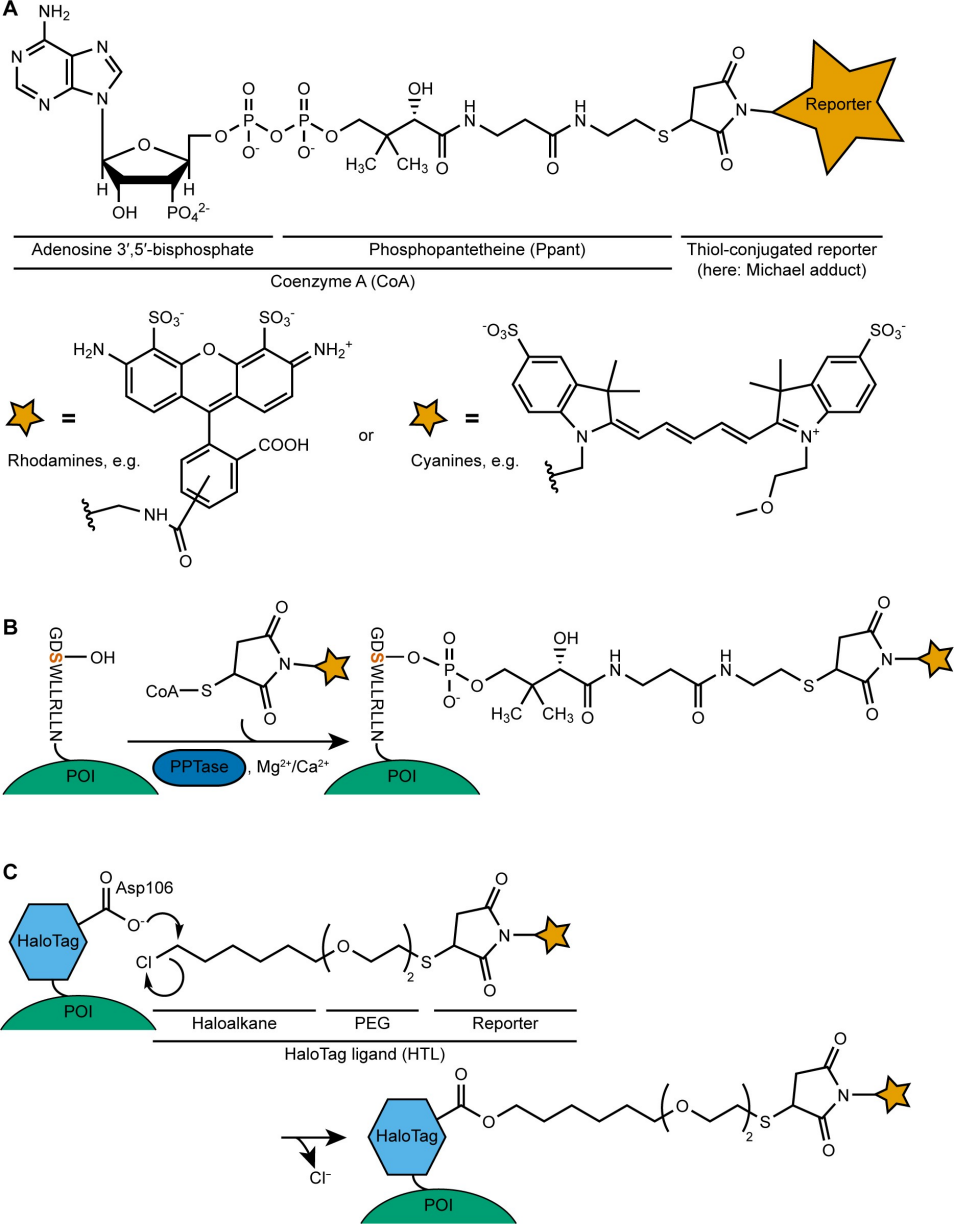
Live-cell labeling strategies described in this work. (A) Structure of a CoA-reporter conjugate [7], and examples of possible reporter dyes, easily attachable through maleimide chemistry to the thiol group of CoA. (B) Labeling of small peptide tags, genetically fused to the protein of interest (POI), mediated through 4′-phosphopantetheinyl transferase (PPTase) enzymes. (C) A HaloTag ligand (HTL), comprising a chloroalkane, polyethylene glycol (PEG) linker units for better access to the catalytic center of the enzyme as well as for improved solubility [8], and a reporter, in this example again coupled via thiol chemistry as in (A).The HaloTag, which can be genetically fused to the protein of interest, is a haloalkane dehalogenase engineered to stably form a reaction intermediate, allowing covalent incorporation of labeled substrates.

One strategy to couple chemical probes to surface proteins appearing particularly attractive is the use of 4′-phosphopantetheinyl transferases (PPTases). The natural role of PPTases is to transfer the phosphopantetheinyl (Ppant) arm of Coenzyme A (CoA, Fig 1A) to carrier proteins, where Ppant acts as prosthetic group [4]. Early applications of this concept were based on genetic fusions of the whole *Escherichia coli* acyl carrier protein (ACP), acting as a substrate for the corresponding PPTase AcpS [5], or of a peptidyl carrier protein (PCP), which is part of a non-ribosomal peptide synthetase complex and a substrate for the PPTase Sfp [6], of *Bacillus subtilis*.

Both PPTases are surprisingly tolerant to using CoA substrates that are derivatized at the sulfhydryl group. This implies that virtually all probes (e.g., fluorophores or biotin) carrying a maleimide, which are often readily commercially available, can be coupled via straightforward Michael addition to the thiol group of CoA (Fig 1A). Furthermore, while Sfp accepts both ACP and PCP as substrates, AcpS prefers ACP [9]. Therefore, orthogonal labeling of two surface proteins, genetically fused to ACP and PCP, respectively, can be achieved. To this end, the cells are sequentially labeled by first incubating with a CoA conjugate (e.g., a “red” dye) together with AcpS as the PPTase enzyme, and secondly a different CoA conjugate (e.g., a “green” dye) together with Sfp as the PPTase.

However, the use of PPTases looked even more attractive when using smaller acceptor moieties or peptides, as they would only minimally disturb the surface protein of interest. The system was further refined by Zhou et al. [7, 10] who, by using phage-displayed peptide libraries, were able to identify genetically encoded short peptide tags (GESPT), replacing the larger ACP and PCP domains, and thereby reducing the size of the required tag fusion to merely 12 amino acids (Fig 1B). These peptide tags are substrates for AcpS and Sfp, respectively, and retain orthogonal reactivity towards the two PPTases, again enabling two-color labeling schemes.

In contrast to the PPTase-dependent systems, the HaloTag (HT) is a 34 kDa self-labeling enzyme (Fig 1C). Based on the haloalkane dehalogenase from *Rhodococcus rhodochrous*, it is also intended for genetic fusion to the protein of interest. Crystal structures were used to guide Los et al. [8] in the design of a stable protein, which becomes trapped in an intermediate, where the alkyl residue remains covalently bound to the enzyme after chloride displacement. Further engineering for higher association rates (see Discussion) yielded the HaloTag, which is capable of covalent conjugation of HaloTag ligands (HTLs), in which a haloalkane is linked to a probe of choice, typically a fluorescent dye.

Several landmark publications have highlighted the importance of reporting negative or (partially) conflicting results in order to prevent unnecessary replication, confirmation bias, selective reporting, and other undesired phenomena of current practices in science dissemination [11–13]. Here, we thus report the observations which highlight possible limitations and drawbacks of the widely applied PPTase-based labeling methods when used on the cell surface. Furthermore, we show a quantitative comparison of PPTase-based and HaloTag-mediated labeling, which so far was not available in the literature and suggests HaloTag labeling as a viable alternative for applications where labeling of unwanted molecules ("non-specific labeling") could lead to erroneous conclusions.

## Results

### Protocol-dependent non-specific coupling of CoA derivatives

**Fig 2:**
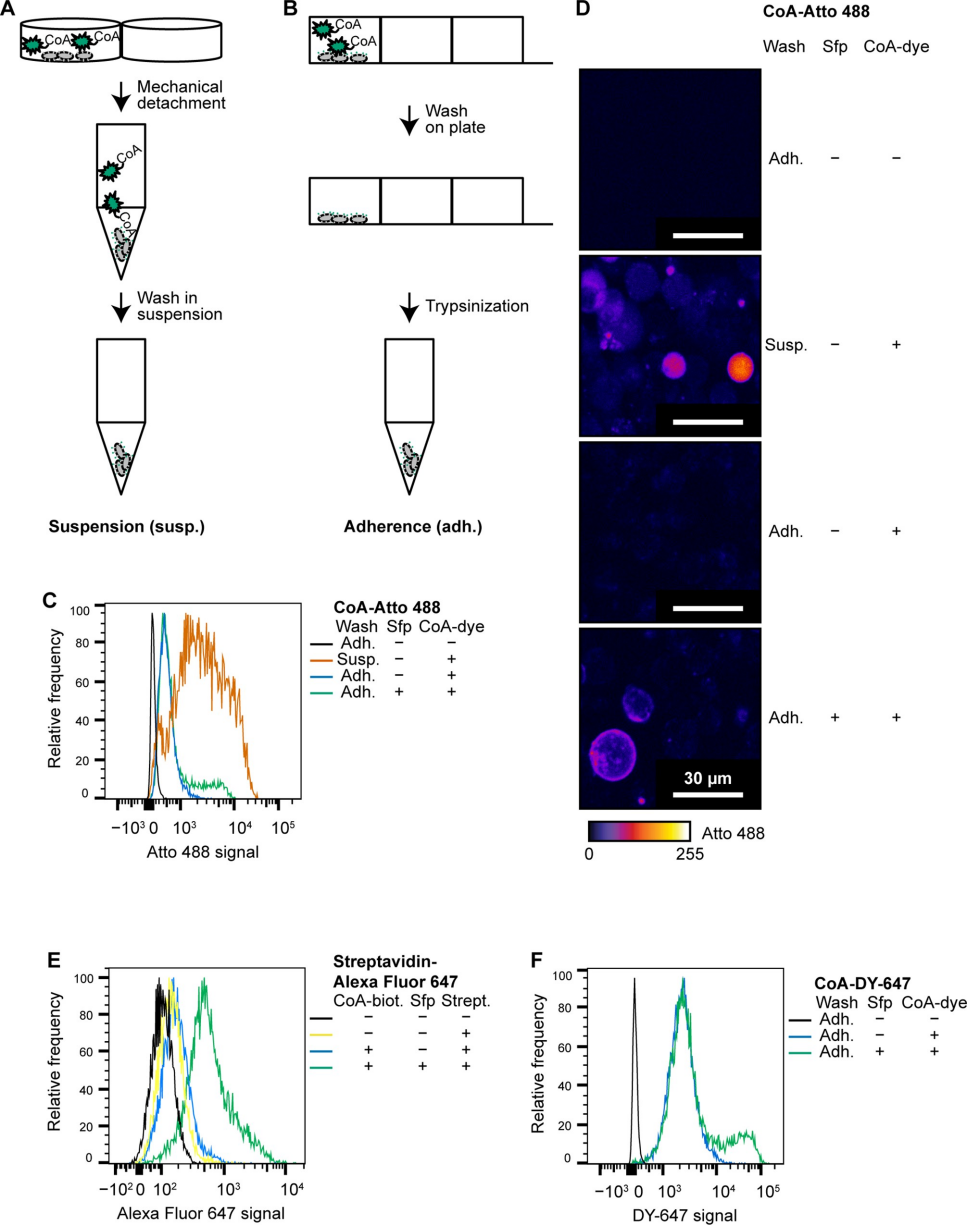
Non-specific staining of cells due to coupling of CoA derivatives to cytoplasmic molecules. (A, B) Schemes illustrating the labeling protocols with wash steps performed either on cells in suspension (A) or on adherent cells (B), respectively. (C) Flow cytometry reveals that non-specific coupling of CoA-Atto488 is, although still present, massively reduced by washing HEK 293 cells while still adherent. (D) Confocal microscopy illustrates homogenous cytosolic stain after washing HEK 293 cells in suspension, indicating cytoplasmic uptake and strong retention of label. Predominantly membranous labeling is observed if CoA-488 is removed while tagged HER2-expressing cells are still adherent. (E) Two-step labeling, in which CoA-biotin on the surface of HEK 293 cells is detected in flow cytometry by streptavidin-Alexa Fluor 647, results only in very limited non-specific staining, in addition to some generic streptavidin binding to the cell surface. (F) CoA-DY 647, a far-red dye, results in significant background binding, irrespective of the optimized wash protocol in flow cytometry.

In our approaches to investigate the dynamics of the receptor tyrosine kinase HER2 in response to various antitumor protein drugs (Stüber et al., *manuscript in preparation*), we needed to follow the receptor in single-molecule tracking experiments at the cell surface, and aimed for a labeling approach with fluorescent dyes. For this purpose, we initially intended to employ the genetically encoded short peptide tag technology with PPTases [7] mentioned above. Therefore, we transiently transfected HEK 293 cells with an N-terminal fusion of the acceptor dodecapeptide S3, a substrate for the PPTase Sfp, to the receptor tyrosine kinase HER2 (ErbB2). This appeared attractive because of the small size of this peptide. However, we observed a strong non-specific staining signal (defined as the fluorescence signal obtained when the Sfp enzyme was omitted from the labeling mix) in initial experiments. Here, after the actual labeling reaction, we washed off the weakly adherent HEK 293 cells using a pipette, and then, afterwards, removed the remaining non-reacted CoA conjugate in solution by several rounds of centrifugation, decanting, and re-suspension in fresh buffer (Fig 2A, Fig 2C). This already hinted at the dyes linked to CoA reacting with other molecules, and then being no longer able to be removed by extensive washing.

Experimental steps subsequent to cell surface protein labeling, such as flow cytometry or single-molecule experiments in high-precision cover slide chambers, frequently require cell detachment. Cell scraping is a routine procedure in cell culture for this purpose, in particular if potential surface protein cleavage by trypsin is a concern, and even recommended for sub-cultivation of certain cell types [14]. However, one might be concerned that the mechanical detachment of cells essentially results in scrape-loading with dye, since scrape-loading has been described as means to introduce even large molecules into cells, probably by transient permeabilization through mechanical force [15]. Even though the cells are washed, micromolar concentrations of dye might remain after labeling. Confocal microscopy clearly showed that the non-specific signal was homogeneously cytosolic (Fig 2D), suggesting that transient permeabilization allows CoA conjugates to enter the cell. Importantly, the dye-carrying PPant arm may easily be irreversibly transferred to cytosolic substrates by cytosolic PPTases, potentially even to the apo-forms of endogenous acyl carrier proteins. The observation that the fluorescent molecules cannot be removed, even by stringent washing, supports the notion that the interaction may be covalent.

When we adapted the protocol such that the cells were carefully washed while still adherent, the non-specific signal was dramatically reduced (Fig 2B, Fig 2C, S1 Fig) for the CoA-Atto488 substrate, and confocal microscopy now showed a specific, membranous stain only in the presence of Sfp (Fig 2D). To support our interpretation that the mechanical detachment led to scrape loading, we also performed a two-step labeling protocol, in which we first coupled CoA conjugated to biotin to the acceptor peptide S3, washed off excess CoA-biotin in suspension, and subsequently detected biotin with fluorescently labeled streptavidin. We reasoned that transfer of streptavidin to the cytoplasm would be minimal, as it is large and hydrophilic and is applied *after* any mechanical stress on the cells. In line with our hypothesis, the low residual non-specific signal was almost exclusively due to well-known non-specific non-covalent binding of streptavidin to cells (Fig 2E). However, because the multivalence of streptavidin can potentially interfere with the unbiased observation of biotinylated surface receptors in single-molecule tracking experiments, or otherwise even activate receptors, this two-step labeling procedure is not a suitable resort. It follows for the PPTase system that its main liability is non-specific coupling to cytosolic acceptors, and to minimize it, CoA-derivatives should be used for which cytosolic delivery is minimal.

Unfortunately, the reduced non-specific cytoplasmic labeling, (due to the improved protocol avoiding mechanical scraping, with washing carried out on the adherent cells) was only observed with CoA-Atto488 and did not transfer to CoA-DY647 (Fig 2F), which also was commercially available until recently. Since our experiments required a second dye in the far-red spectral range, we synthesized a series of cyanine- and rhodamine-CoA conjugates according to established protocols [9]. However, all of them showed non-specific staining, defined as fluorescence observed in the absence of tagged HER2 and/or absence of PPTase. We hypothesize that the higher ‘stickiness’ of many of these far-red dyes causes them to be internalized passively more efficiently, even if they are per se cell-impermeable, and that they can then be subsequently covalently trapped by the same enzymatic mechanism. Therefore, none of the red dye-CoA conjugates were of use for our applications. We also tested several further factors potentially influencing the relative non-specific signal—e.g., the number of washes, the used cell lines, fixation methods, or cell health (comparing dead and live cells)—to no avail. This means that the trapping of CoA-based dyes by cytoplasmic PPTases, compounded by the non-negligible transfer of the dyes to the cytoplasm at the concentrations needed (see below) prevents the clean labeling of surface proteins, which would be essential for their biophysical study in cells.

### Alternatives to PPTase-based technologies: HT labeling

**Fig 3:**
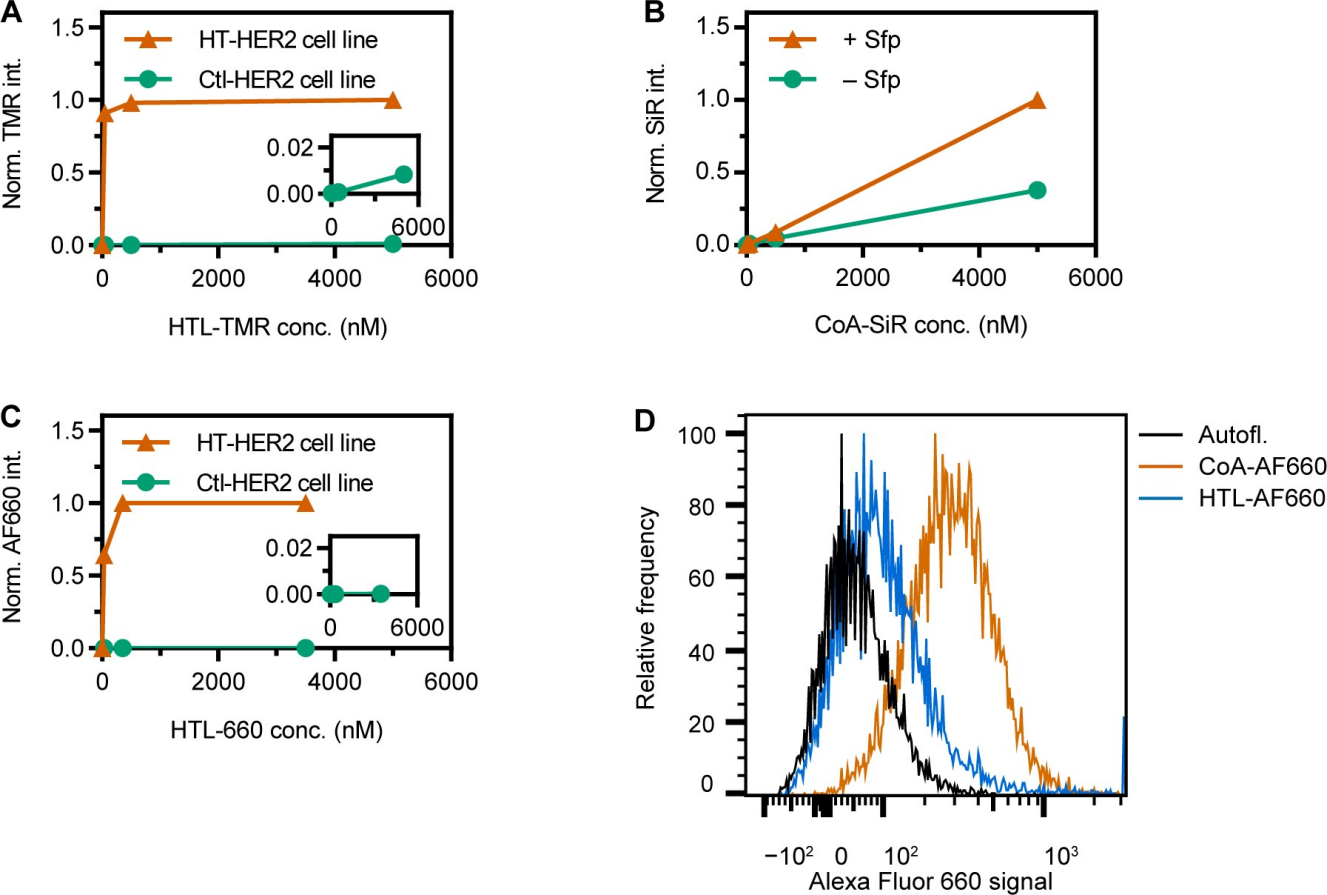
Comparison of PPTase and HaloTag (HT) labeling. (A) Using cell-permeable HTL-TMR, saturation is reached by labeling for 15 min with nanomolar concentrations with high specificity (vermillion curve), as very low signal is found in the absence of HT fusion (bluish green curve). (B) In contrast, saturation is not reached by 5 μM of cell-permeable CoA-SiR, even after 30 min incubation with 1 μM Sfp enzyme (vermillion), with about 30% of the signal being non-specific (in the absence of enzyme, bluish green). (C) A HaloTag ligand (HTL) based on Alexa Fluor 660 results in extremely low non-specific signal (absence of HaloTag, inset, bluish green), while the kinetics are only slightly less favorable than those for HTL-TMR (vermillion). (D) Flow cytometry of HEK 293 cells (no HT, no Sfp enzyme) (black), compared with the same cells after non-specific labeling after 15 min incubation with 15 μM of the respective HTL (blue) or CoA derivative (vermillion).

In search for a suitable cell surface protein labeling technology, we then turned to the HaloTag (HT) system. We found that specific labeling of surface HER2 was possible with a commercially available, cell-permeable ligand containing tetramethylrhodamine (TMR). Because the intrinsic reaction kinetics are much more rapid, only low dye concentrations are needed to achieve labeling in the same time period, and thus less dye is available to enter the cell, where it furthermore is not retained by any cell-bound reactant. Saturation was reached in the low nanomolar range on a HEK 293-derived cell line expressing high levels of HT-HER2 (Fig 3A). Under these conditions, non-specific staining (defined as fluorescence intensity recorded for a cell line not expressing the HT-fusion) was still sufficiently low even at up to 5 μM total dye-haloalkane conjugate.

We produced a similar ligand for the CoA system by coupling the cell-permeable silicon-rhodamine (SiR)-carboxyl maleimide to CoA. This dye (not conjugated to CoA) had been shown to intrinsically bind only very little to cellular components in a non-specific manner [16]. However, as a CoA-conjugated substrate, 5 μM of this substrate were not sufficient to saturate HEK 293 cells which, after transfection with an appropriate vector, displayed acceptor peptide tags fused to HER2 on the surface. The signals increase linearly over a wide range (Fig 3B), typical for a non-specific, non-saturable component.

Since we obtained exceptionally specific labeling using a HT ligand (HTL) containing the far-red fluorescing Alexa Fluor 660 (HTL-660, Fig 3C), we also coupled Alexa Fluor 660 to CoA, and could thus directly compare the relative influence of the CoA and chloroalkane moieties on non-specific dye accumulation. The results (Fig 3D, S2A Fig) demonstrated that even using a dye which is essentially inert to cellular uptake, CoA-based labeling strategies are inferior to the HT system as they give rise to higher non-specific labeling. Very similar data were obtained with an even lower dye concentration in an independent experiment (S2B Fig).

## Discussion

Many biochemical experiments to study the behavior of surface receptors rely on fluorescence, when their association, internalization and degradation is to be studied, e.g., as a consequence of externally added ligands. Many of these experiments, in particular super-resolution microscopy techniques, for example, direct stochastic optical reconstruction microscopy (dSTORM), single-molecule co-tracking, or pulse-chase protocols, require either extremely photostable dyes and/or labeling with more than one fluorophore, often with spatial and/or temporal resolution. In all these cases, the use of fluorescent proteins is inadequate, and a small-molecule fluorophore needs to be attached to the protein of interest on the cell surface. Clearly, the exclusive labeling of the protein of interest is of utmost importance.

Fluorophores non-specifically bound to other cellular components will almost inevitably affect the observed biophysical parameters, such as diffusion coefficients. Therefore, the degree of such non-specific labeling should not only be quantitatively assessed but also minimized for each experimental system. The PPTase system appears attractive, especially when using it with short acceptor peptides, as it would be expected to disturb the receptor under study only minimally.

For PPTase-based labeling of a carrier protein or acceptor peptide (fused to the membrane protein of interest) to be judged as "specific" requires that the signals from cells (not expressing the tagged protein of interest), which have been incubated with the respective CoA-conjugate, is equal to the autofluorescence of completely untreated cells. We screened the 119 publications that cite (at the time of writing according to the Web of Science) the original article [7] describing the peptide tag labeling system we employed, whether they mention such a specificity control by microscopy or flow cytometry. While many of these are review articles, it is still noteworthy that we found only one research article [17] mentioning non-specificity controls in flow cytometry; however, in this work, the auto-fluorescence signal necessary for comparison is not reported, and an intracellular CoA-Atto647N signal is evident in the microscopy images shown. Also, highly specific labeling in the system employed by Humpert et al. [17] would actually be rather surprising, as these authors used a CoA conjugate of Atto647N, a dye notorious for non-specific cell binding [18, 19], due to its positive charge and hydrophobicity. Another study [20] demonstrated specific surface protein labeling for single-molecule imaging mediated by the short peptide acceptor tags. Notably, this required a two-step procedure, in which biotin was detected by streptavidin-quantum dot conjugates, with the same advantage (no detection of intracellular CoA-biotin) as described above for our control experiments with streptavidin-Alexa Fluor 647, yet with the caveat of possible oligomerization induced by the detection system. We thus conclude that the absence of specificity controls in the 119 publications may be mostly based on an implicit belief by the authors of the inherent specificity of the system—which it undoubtedly can show in the absence of cellular PPTases, or when access to them is strictly prevented.

PPTase labeling is and remains a versatile and attractive technique which allows unique applications. With the exception of the incorporation of unnatural amino acids, which itself presents major technical challenges, to our knowledge, no other technique is as "non-invasive” based on the small size and in vivo compatibility of the PPTase acceptor peptide tags [21]. However, we demonstrated here that the specificity of PPTase tag systems is intrinsically limited by the lack of orthogonality in whole cells—due to the ubiquitous role of CoA and the Ppant moiety in metabolism of pro- and eukaryotes—since CoA derivatives, once having reached the cytosol, will react and remain there, as strongly suggested by the cytosolic staining observed in the absence of the PPtase enzyme (Fig 2D). We would like to add that we cannot exclude a role in other nucleophiles in retaining the CoA derivatives in the cytosol. In summary, researchers considering which covalent labeling methods to choose need to be aware of the limitations of the PPTase methods, which we have demonstrated here for the first time by quantification of the non-specific signals.

We conclude that for delicate applications, where non-specific labeling, and therefore dye exposure (incubation time and concentrations) must be reduced to a minimum, self-labeling enzymes are likely more suitable. Regarding reaction kinetics, a very fast second-order association rate constant is required, since the dye needs to be applied at low concentrations (to avoid unspecific cellular uptake and unspecific binding), and very long reaction times may alter the cellular state. Our example, the HT, has a rather fast bimolecular rate [8], much faster than all PPTase-based systems at substrate- and enzyme-concentrations that can realistically be reached, since PPTase catalyzes a sequential, Mg^2+^-dependent bi-substrate reaction [22, 23]. It thus shows much slower kinetics at the achievable concentrations in cellular labeling. This is because, under conditions of live cell labeling, both the CoA conjugate (typically, ≤5 μM, limited by the non-specific signal increasing with concentration), as well as the surface protein (typically, ≤1 nM, obviously depending on surface expression levels), are present in sub-saturating concentrations [7, 10, 24, 25]. Therefore, at concentrations and after incubation times where non-specific labeling is acceptable, the reaction will often be incomplete (Fig 3B), and further increasing the amount of PPTase enzyme eventually becomes uneconomical. It may also be noted that some of the components (enzymes and CoA-conjugates) for PPTase labeling are no longer commercially available [26]. Nonetheless, in-house production of them is rather straightforward [7].

Despite its advantages, it needs to be stressed that the Halo "tag" is in reality an enzyme of 34 kDa, and in a given biological system it needs to be established whether this additional domain might interfere with protein function. The ACP tag (8 kDa) is of an intermediate size, while small acceptor peptides (1 kDa) are clearly the least disturbing entities. For completeness, we would like to mention that other peptide ligases, with the tags ranging from 5–116 amino acid residues, have been used for similar purposes [27–29].

Two-color labeling, an attractive feature of orthogonal acceptor peptide tags, may also be obtained by combining HTged and SNAP-tagged [30] enzymes, and while they may not possess the same substrate promiscuity as PPTases, a wide range of functional ligand conjugates for both systems has been reported [16, 31, 32]. It should be noted in this context that the HT favors rhodamines, in particular TMR, by design [8]; however, we have also obtained good results with cyanines, e.g., Alexa Fluor 647, which may be more suited for certain applications than the fast-bleaching Alexa Fluor 660, such as localization microscopy. Furthermore, the HT also allows intracellular labeling with little background (Fig 3A), which is impossible for CoA-dependent systems because of the abundance of cytoplasmic PPTases, and this HT system has thus allowed development of an assay for surface protein internalization and degradation based on sequential extracellular and intracellular labeling [33].

### Limitations

In the present manuscript, we demonstrated that for certain, delicate applications, the specificity of enzymatic live cell labeling based on PPTases and CoA derivatives is inherently insufficient. Our goal is to prevent unnecessary replication of our results and of our optimization efforts by other researchers. We point out alternative methods which work very well in this context. While we only studied one acceptor peptide, the limitations of the system largely seem to arise from the CoA conjugates and are thus independent of the PPTase and acceptor peptide/carrier protein used.

Nonetheless, it may still be possible to overcome these limitations of the PPTase system by optimizing several components. We did not investigate the 8-kDa carrier proteins, which are essentially equivalent to the natural PPTase substrates, as alternatives to the small peptide tags. For example, for the ACP [5] tag, better kinetic parameters than for the dodecapeptides have been reported, thus potentially allowing to lower the CoA-dye concentration such that non-specific labeling becomes acceptable. We furthermore only included one benchmark—the self-labeling enzyme HT and the corresponding ligands—in our experiments and stringently showed its superiority for only one dye, yet this system clearly performed excellently for our purposes.

Our observations obviously also do not apply to any in vitro application of PPTases, for which countless successful examples can be found in the literature. Therefore, we want to stress that we do not wish to implicitly discourage considering CoA-based cell labeling protocols. It is important for researchers, however, to understand the sources of nonspecific signals in PPTase based systems.

A comprehensive quantitative assessment of all currently relevant labeling techniques with regards to their specificity, as well as the attainable degree of labeling, would thus be desirable and assist researchers in the choice of their experimental system, yet would be obviously beyond the scope of the present study.

## Materials and methods

### PPTase labeling

For PPTase labeling experiments and comparison of protocols (Fig 2A–2D, Fig 2F), we cloned HER2 (ErbB2, Mammalian Gene Collection, GenBank accession number BC156755.1) into a pcDNA3.1(−)/*myc*-His(−) B vector (Thermo Fisher, cat. no. V855-20), with the S3 tag [7] at the N-terminus, connected by a G_4_S-linker sequence, to yield pcDNA-S3-HER2. Note that we used the tag designated “S3” in the manuscript by Zhou et al. [7] because, according to the supplementary experimental section of this paper, primers leading to the attachment of this peptide (rather than the variant designated “S6” and mentioned in the main text) appear to have been used in the preparation of their final EGFR fusion constructs. It should be noted however, that the non-specific signal is completely independent of the peptide tag actually used, as it was also observed with non-transfected cells (Fig 3D).

HEK 293 cells (ATCC, cat. no. CRL-1573) were seeded at a density of 2∙10^5^ cells per well in 24-well tissue culture plates (Nunc, cat. no. 353047), and, on the next day, transiently transfected with pcDNA-S3-HER2 using the TransIT-293 (Mirus Bio, cat. no. 2700) transfection reagent according to the instructions by the supplier. One day later, the medium was replaced by 300 μl fresh medium per well, supplemented with 10 mM MgCl_2_ and containing 5 μM of the respective CoA-conjugate (NEB, CoA-Atto488, cat. no. S9348, CoA-DY647, cat. no. S9350), either with 1 μM Sfp (NEB, cat. no. 9302) or without it (for non-specificity controls), and incubated for 30 min at 37°C (in a 5% CO_2_ atmosphere).

For the procedure in which cells were, after labeling, first detached and the dye subsequently removed by repeated re-suspension (Fig 2A), 500 μl Dulbecco’s PBS (DPBS) were added and the weakly adherent cells detached and suspended by vigorous mixing with a 1000 μl micropipette. The cell suspension was then transferred to 1.5 ml microcentrifuge tubes, washed three times in 700 μl DPBS each by centrifugation and re-suspending, and finally re-suspended in 500 μl DPBS and stored on ice until measurement.

For the procedure in which cells were washed while still adherent (Fig 2B), cells were, after labeling, washed carefully four times with 700 μl warm medium, then detached with 200 μl trypsin-EDTA solution, 800 μl complete medium was added, the samples then centrifuged and re-suspended in 500 μl DPBS, and stored on ice until measurement.

### Two-step PPTase-streptavidin labeling

Two-step labeling experiments (Fig 2E) were performed with a HEK 293-derived cell line stably expressing a S3 tag fusion construct similar to that used for transient transfections (pcDNA-S3-HER2, see above), however, containing HER3 (ErbB3) with a C-terminal sfGFP [34] fusion to enable single-clone selection by fluorescence-activated cell sorting. Cells were seeded in 24-well plates and two days later labeled as above, however with 5 μM CoA-biotin (NEB, cat. no. S9351), then the cells were washed twice with complete medium while still adherent as detailed before. After detachment, cells were re-suspended into a 50 nM solution of streptavidin coupled to Alexa Fluor 647 (Thermo Fisher, cat. no. S21374) in DPBS supplemented with 1% (w/v) bovine serum albumin and 0.1% sodium azide (PBSBA), and incubated for 35 min on ice. Afterwards, cells were washed once by centrifugation and re-suspension in 800 μl PBSBA, then once in 800 μl ice-cold PBS, and then fixed in 1 ml 2% (w/v) solution of paraformaldehyde in DPBS for 20 min at room temperature. Finally, cells were washed once in 800 μl PBSBA, re-suspended in 500 μl PBSBA, and stored at 4°C until measurement.

### Coupling of and labeling with CoA-SiR

SiR-carboxyl maleimide was conjugated to Coenzyme A (tri-lithium salt, Sigma, cat. no. C3019) according to established protocols [9], purified via HPLC (S3A Fig), and the product confirmed by ESI(+)-MS. PPTase labeling was performed as described above, however, with CoA-SiR concentrations varying as indicated, and HEK 293T/17 cells (ATCC, cat. no. CRL-11268) were used to obtain high transfection efficiencies.

### HaloTag labeling

For HTL titration experiments, we used a HEK 293-derived cell line stably expressing a construct in which the HaloTag7 is N-terminally, and sfGFP [34] is C-terminally fused to HER2. This was obtained by transfection with a plasmid containing the aforementioned construct in a modified pSems vector (kind gift by Dr. Stephan Wilmes and Prof. Jacob Piehler, University of Osnabrück). A cell line stably expressing a similar construct, however, with an (in this case irrelevant) S3 peptide tag N-terminally fused, instead of the HT, was used as non-specificity control.

For labeling experiments, cells were seeded in 24-well cell culture plates in 1.5 ml medium as above, and, two days later, 1.1 ml of the medium was removed and a 5-fold stock solution of the HTLs (Promega, cat. nos. G8252 and G8472) added to yield the indicated concentrations. After incubation at 37°C (in a 5% CO_2_ atmosphere) for 15 min, cells were washed three times with 700 μl of warm complete medium, and the cells were further incubated in 700 μl fresh complete medium for 30 min. Afterwards, cells were detached with 400 μl trypsin-EDTA, 800 μl complete medium was added, the samples centrifuged, washed once in 1 ml PBS, re-suspended in 1 ml 2% (w/v) paraformaldehyde in DPBS, and incubated for 20 min at room temperature. Subsequently, cells were washed once in 1 ml DPBS, re-suspended in 500 μl PBSBA, and stored at 4°C until measurement.

### Direct comparison of and CoA-AF660 and HTL-AF660

CoA-AF660 was obtained by coupling Alexa Fluor 660 C_2_ maleimide (Thermo Fisher, cat. no. A20343) to CoA [9], HPLC-purified (S3B Fig), and its integrity confirmed by mass spectrometry. HEK 293 cells (not expressing any tag) were seeded, labeled with 3.5 μM of either CoA-AF660 or HTL-AF660, respectively, for 20 min, and processed in parallel, as detailed above for the HaloTag labeling. However, after cell detachment, cells were washed three times in 700 μl PBSBA before final re-suspension in 500 μl DPBS supplemented with 0.1% (w/v) sodium azide.

### Flow cytometry

Flow cytometry data were acquired on FACS Canto II or LSR II Fortessa (BD) instruments, and analyzed with FlowJo 10.4 (FlowJo).

### Microscopy

Imaging was performed on a SP5 confocal laser scanning microscope (Leica) and data processed using Fiji [35].

### Data coverage

Experiments were repeated fully independently and only the results of one of the experiments shown where appropriate (e.g., for flow cytometry histograms and microscopy images). Where suitable, independent repetition data alongside with a reduced representation of the data in the main text figures are shown in the Supplementary Figures (S1 Fig, S2 Fig). Number of replications for each of the main text figure panels: Fig 2C, Fig 2D: 3 independent replications (S1 Fig); Fig 2E: 3 independent replications; Fig 2F: 2 independent replications; Fig 3A, Fig 3C: 2 independent replications; Fig 3B: 2 independent replications; Fig 3D: 2 independent replications (S2 Fig).

## Supporting information

S1 Fig Independent replications of protocol comparisons. The experiment shown in Fig 2C, for which the full single-cell histograms are shown, was repeated independently three times, the median fluorescence intensity normalized to the signal of the unlabeled control, and the mean of the median fluorescence intensity plotted together with the standard error of the mean.
(TIF)

S2 Fig Quantitative comparison of non-specific labeling for the PPTase and HaloTag systems. Side-by-side labeling experiments were performed with native HEK 293 cells not expressing any tagged membrane protein (i.e., signals could only arise due to non-specific labeling reactions) using either the PPTase or HaloTag labeling protocols. (A) Median fluorescence intensities of the data shown in Fig 3D, which were obtained using 20 μM of the respective dye conjugates. (B) Fully independent reproduction of the observation in (A), using 1.6 μM of the dye conjugates.
(TIF)

S3 Fig Reversed-phase high performance liquid chromatography analyses of CoA conjugates. (A) Free SiR-maleimide (vermillion) and the CoA-SiR conjugation mixture (bluish green) were quenched by addition of β-mercaptoethanol in excess and analyzed using an optimized gradient of acetonitrile in 0.1% (v/v) trifluoroacetic acid (MeCN/TFA, B) in ultrapure water. The black line below the peak indicates the collected volume fraction. (B) Analytical run of free Alexa Fluor 660 maleimide (vermillion), free CoASH (reddish purple), and the reaction mix (bluish green). The conjugate was subsequently isolated using a shallower gradient to achieve improved separation from the free dye. All analyses and small-scale preparations ((A) and (B)) were run on an Xterra RP-C18 HPLC column (Waters).
(TIF)

## Abbreviations

PPTases: 4′-phosphopantetheinyl transferases
Ppant: phosphopantetheinyl
ACP: acyl carrier protein
PCP: peptidyl carrier protein
GESPT: genetically encoded short peptide tags
HT: HaloTag
HTL: HaloTag ligand
TMR: tetramethylrhodamine
