# Supplementary figures and images for: Labeling surface proteins with high specificity: Intrinsic limitations of phosphopantetheinyl transferase systems
Source: PLoS One. 2019 Dec 19;14(12):e0226579. doi: 10.1371/journal.pone.0226579 (PMC6922365; doi:10.1371/journal.pone.0226579)

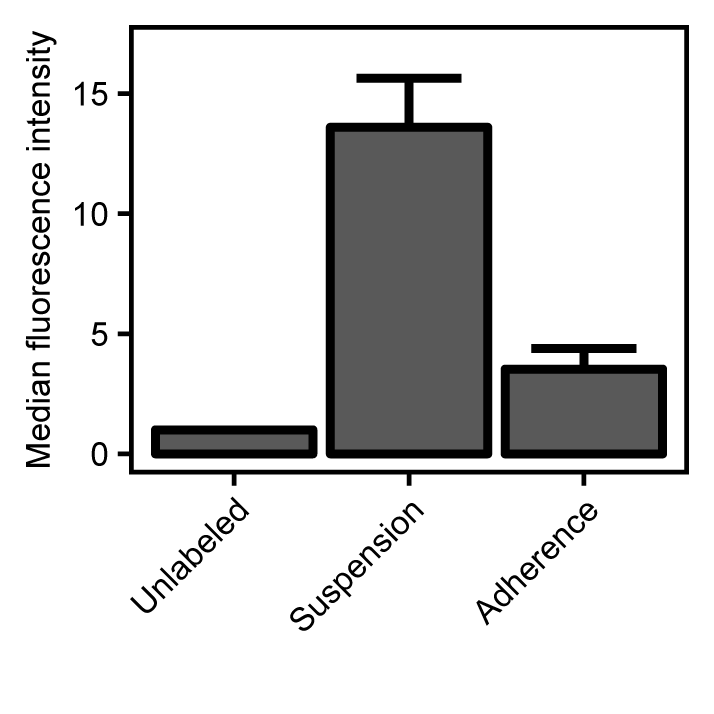

Supplement: S1 Fig — The experiment shown in Fig 2C, for which the full single-cell histograms are shown, was repeated independently three times, the median fluorescence intensity normalized to the signal of the unlabeled control, and the mean of the median fluorescence intensity plotted together with the standard error of the mean. (TIF) [file pone.0226579.s001.tif]

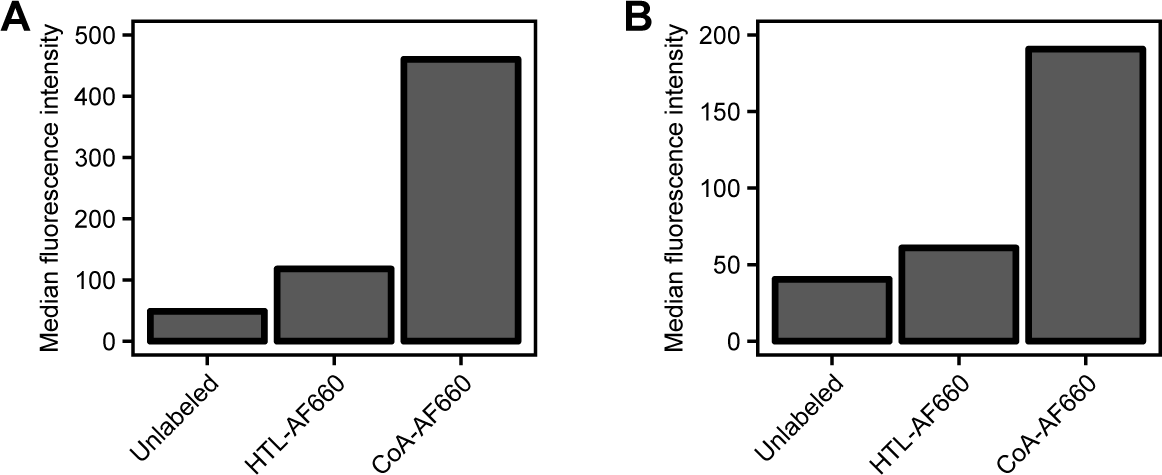

Supplement: S2 Fig — Side-by-side labeling experiments were performed with native HEK 293 cells not expressing any tagged membrane protein (i.e., signals could only arise due to non-specific labeling reactions) using either the PPTase or HaloTag labeling protocols. (A) Median fluorescence intensities of the data shown in Fig 3D, which were obtained using 20 μM of the respective dye conjugates. (B) Fully independent reproduction of the observation in (A), using 1.6 μM of the dye conjugates. (TIF) [file pone.0226579.s002.tif]

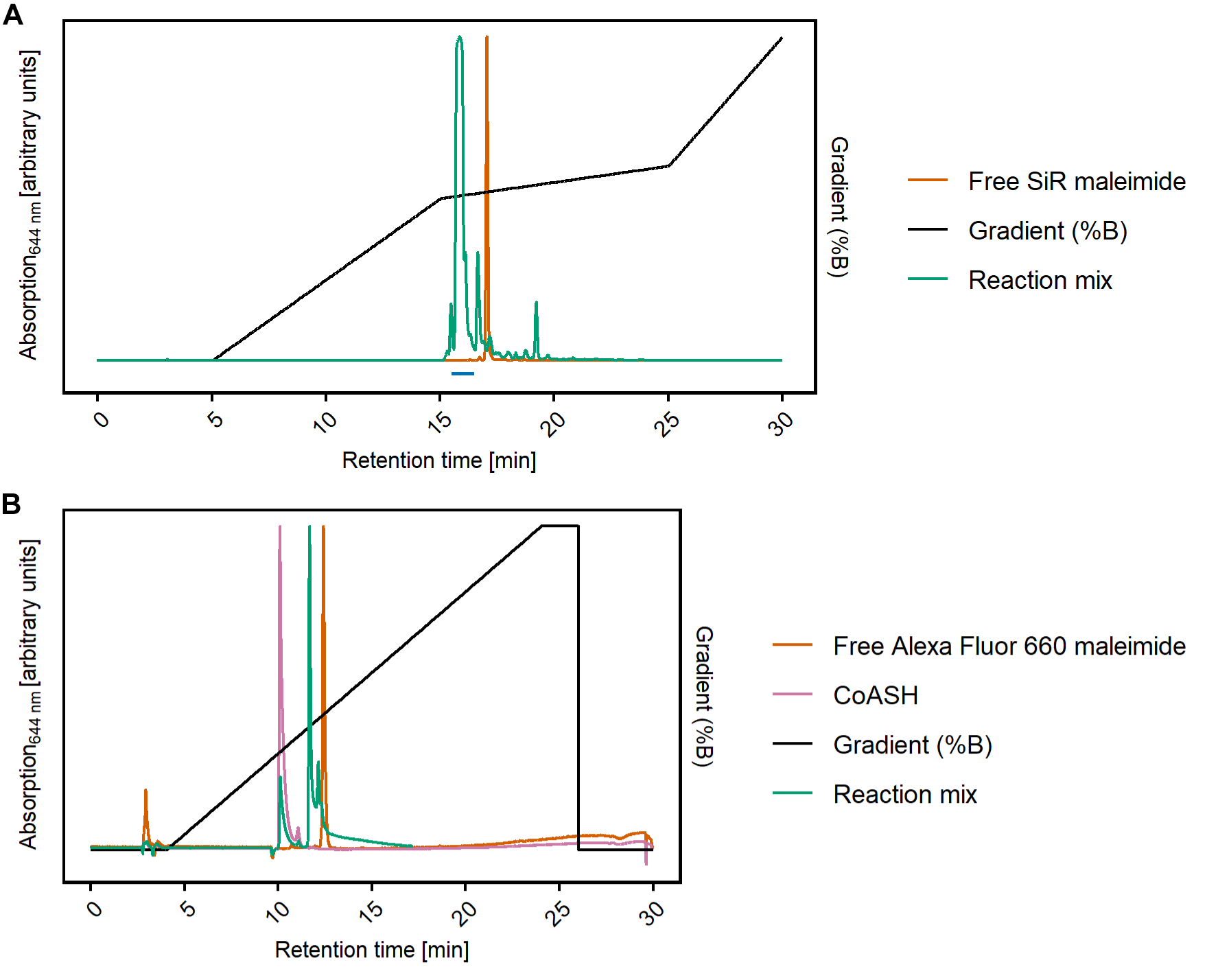

Supplement: S3 Fig — (A) Free SiR-maleimide (vermillion) and the CoA-SiR conjugation mixture (bluish green) were quenched by addition of β-mercaptoethanol in excess and analyzed using an optimized gradient of acetonitrile in 0.1% (v/v) trifluoroacetic acid (MeCN/TFA, B) in ultrapure water. The black line below the peak indicates the collected volume fraction. (B) Analytical run of free Alexa Fluor 660 maleimide (vermillion), free CoASH (reddish purple), and the reaction mix (bluish green). The conjugate was subsequently isolated using a shallower gradient to achieve improved separation from the free dye. All analyses and small-scale preparations ((A) and (B)) were run on an Xterra RP-C18 HPLC column (Waters). (TIF) [file pone.0226579.s003.tif]
